# Determinants of government HIV/AIDS financing: a 10-year trend analysis from 125 low- and middle-income countries

**DOI:** 10.1186/1471-2458-13-673

**Published:** 2013-07-19

**Authors:** Carlos Ávila, Dejan Loncar, Peter Amico, Paul De Lay

**Affiliations:** 1 Abt Associates, 4550 Montgomery Ave, Suite 800 North, Bethesda, MD 20814, USA; 2 Joint United Nations Programme on HIV/AIDS (UNAIDS), 20 Avenue Appia, Geneva, Switzerland; 3 Research Triangle Institute International, Waltham, Massachusetts, USA

## Abstract

**Background:**

Trends and predictors of domestic spending from public sources provide national authorities and international donors with a better understanding of the HIV financing architecture, the fulfillment of governments’ commitments and potential for long-term sustainability.

**Methods:**

We analyzed government financing of HIV using evidence from country reports on domestic spending. Panel data from 2000 to 2010 included information from 647 country-years amongst 125 countries. A random-effects model was used to analyze ten year trends and identify independent predictors of public HIV spending.

**Results:**

Low- and middle-income countries spent US$ 2.1 billion from government sources in 2000, growing to US$ 6.6 billion in 2010, a three-fold increase. Per capita spending in 2010 ranged from 5 cents in low-level HIV epidemics in the Middle East to US$ 32 in upper-middle income countries with generalized HIV epidemics in Southern Africa. The average domestic public spending per capita was US$ 2.55. The analysis found that GDP per capita and HIV prevalence are positively associated with increasing levels of HIV-spending from public sources; a 10 percent increase in HIV prevalence is associated with a 2.5 percent increase in domestic funding for HIV. Additionally, a 10 percent increase in GDP per capita is associated with an 11.49 percent increase in public spending for HIV and these associations were highly significant.

**Conclusion:**

Domestic resources in low- and middle-income countries showed a threefold increase between 2000 and 2010 and currently support 50 percent of the global response with 41 percent coming from sub-Saharan Africa. Domestic spending in LMICs was associated with increased economic growth and an increased burden of HIV. Sustained increases in funding for HIV from public sources were observed in all regions and emphasize the increasing importance of government financing.

## Background

Over the past 10 years, the world has seen a dramatic scale up of funding in order to combat HIV worldwide. In addition to their domestic response, countries have experienced various levels of bilateral support, primarily from the U.S. President's Emergency Plan for AIDS Relief (PEPFAR), the Organization for Economic Co-operation and Development (OECD) countries and also from multilateral mechanisms such as The Global Fund to Fight AIDS, Tuberculosis and Malaria (GF), the United Nations system and UNITAID. Some countries, specifically in sub-Saharan Africa (SSA) are highly dependent on donor funding which raises concerns about the sustainability of their domestic HIV response [1]. The economic crisis has created widespread concerns that funding shortages will have an adverse impact on public spending affecting the national resources devoted to HIV.

Global spending on HIV is increasing; it is up 11% in 2011 over 2010 at US$ 16.8 billion. International assistance is essentially flat and some donor countries are reducing their funding [2]. Because of the scarcity of health funding, it is important to implement robust financial tracking mechanisms to monitor political commitments and understand the funding trends in the health sector. This will allow policy makers and countries to evaluate their spending patterns and strategically scale up effective interventions with high impact as well as planning for future demand without interruptions to supplies of antiretroviral treatment (ART) and HIV staffing. This paper seeks to understand the independent predictors of domestic spending on HIV between 2000 and 2010 and to examine regional HIV spending trends.

## Methods

### Data sources

In order to evaluate HIV spending, a dataset was constructed by combining country reports that included the total amount of domestic spending at the country level. The domestic/public HIV spending dataset was constructed from the national data reported for United Nations General Assembly Special Session (UNGASS) report indicator number 1 based on the National AIDS Spending Assessment (NASA). The AIDS resource tracking methodology and definitions are explained elsewhere [3,4]. Domestic-public spending on HIV is the amount of funding from a country’s national resources that has been allocated and spent on HIV specific activities, human resources and infrastructure. HIV public-domestic expenditures encompasses all funds that are spent inside of the country from government sources [4]. We excluded domestic private and international sources of funding.

### Research questions

We identified independent predictors of domestic/public spending; we focused the analysis on several plausible variables using existing literature, that may influence a government’s decision to invest in HIV, including the national economy expressed as Gross Domestic Product (GDP) per capita, HIV prevalence and governance indicators [1]. It is hypothesized that as HIV prevalence increases in a country, the domestic/public spending will also increase. This response is explained by the increased priority being given to HIV as it becomes a more significant issue in the country’s population. Due to the vast difference in prevalence across regions, regional variables were employed to account for this variation. Additionally, a country’s level of GDP per capita is also hypothesized to lead to increased investment in domestic/public spending on HIV. It is assumed that spending on HIV is a normal good; this means that as income increases so will the domestic investment in the country’s HIV spending. Governance is another important area to measure as well as the burden of other diseases in the country. It is hypothesized that countries with more stable and less corrupt governments will invest more funding in HIV. For the purposes of this paper we measured these concepts using the World Bank’s governance indicators [5].

### Analytical strategy

The panel consisted of 678 country years of observations covering a span of 10 years and 129 countries. 512 of these data points were reported and validated from country reports. The average number of observations per country was 4.6 and the average domestic public spending per capita was US$ 2.55. The number of independent country observations ranged from 17 in 2000 to 101 in 2008. The final regression model included 647 country years and 125 countries due to the exclusion of outliers. The regression diagnostic tests (leverage, Cook's distance, dfbeta) indicated that Namibia, South Africa, Botswana and Swaziland were outliers and they were excluded from the model.

The independent variables that were used in the analysis at the country level were: GDP per capita, HIV prevalence, an array of governance indicators and regional variables. HIV prevalence is the percentage of people living with HIV. The governance variables included measures of accountability, corruption, government efficiency, political stability, regulatory quality and rule of law. Because these variables were highly correlated we used only political stability to represent the overall concept of governance. GDP per capita and HIV prevalence were log transformed to normalize the residuals and correct skewness and kurtosis. The dependent variable was the domestic public spending per capita on HIV; this variable was also log transformed.

Panel data is likely to violate the Gauss Markov assumptions of independence; therefore, several models were tested—both random and fixed effects models with clustered standard errors. The Hausman and Sargan-Hansen tests confirmed the random effects model was the best specification. The projections are not intended to forecast domestic/public spending on HIV far into the future, due to the instability of public and donor funding, but to show current and past trends.

## Results

Low- and middle-income countries spent a cumulative total of US$ 43.5 billion from domestic public sources between 2000 and 2010. Out of this total; 35 percent was spent in sub-Saharan Africa (US$ 12.9 billion), 32.8 percent in Latin America (US$ 12.1 billion), 12 percent in South-East Asia (US$ 4.42 billion) and 9.5 percent in Eastern Europe and Central Asia (US$ 3.52 billion). Total domestic spending in low-and middle-income countries showed a relative increase of 314 percent during the ten-year period.

### Descriptive statistics and panel sample size

Table 1 shows an overview of the domestic/public spending, HIV prevalence and GDP per capita. The mean of selected independent variables and the dependent variable are shown by region, due to the large amount of variation that occurs across 145 countries with very different spending priorities and epidemiological profiles. Clearly sub-Saharan Africa (SSA) has much higher domestic/public spending as well as a much higher prevalence rate than the other regions. These variations between regions are important differences that are accounted for in the model.

**Table 1:** Descriptive statistics

**Variable**	**SSA**	**LAC**	**Other**	
			**countries**	
	Mean	Mean	Mean	Sample Size
	(SE)	(SE)	(SE)	(SSA, LAC, Other)
Domestic/Public Spending per capita US$	5.52	1.88	1.07	208, 154,
	(17.33)	(1.61)	(3.16)	314
HIV Prevalence	6.97	0.585	0.496	418, 187,
	(7.12)	(.332)	(.617)	616
GDP per capita US$	2,023	4,538	3,884	435, 187,
	(2917)	(2859)	(3561)	787

*Standard errors indicated by ().

## Model specification and independent predictors of domestic public spending

The key estimation results from the model including prevalence, GDP per capita, a variety of governance indicators and regional controls are reported below in Table 2. The final prediction model (Table 2, model 4) was a random effects model taking the natural logarithm of HIV prevalence, the natural logarithm of GDP per capita and including regional controls for sub-Saharan Africa (SSA) and Latin America (LAC). The coefficients from the fixed and random effects model were similar in magnitude. Due to the log-log specification in the model the results can be interpreted as elasticities. Therefore, a 10 percent increase in HIV prevalence is associated with a 2.5 percent increase in domestic/public funding for HIV. Additionally, a 10 percent increase in GDP per capita is associated with an 11.49 percent increase in domestic/public spending for HIV. All of the variables in the prediction model (Table 2, model 4) were highly significant at the .001 percent level. We found that political stability was not significantly associated with domestic/public spending on HIV.

**Table 2:** Domestic/public expenditures on HIV and predictors

	**Fixed effects model**		**Random effects model**	
	(1)	(2)	(3)	(4)
VARIABLES	Ln(domestic public per capita)	Ln(domestic public per capita)	Ln(domestic public per capita)	Ln(domestic public per capita)
Ln(Prevalence)	0.073	0.077	0.247*	0.252*
	(.230)	(.212)	(.077)	(.076)
Ln(GDP_cap)	1.201*	1.268*	1.090*	1.149*
	(.213)	(.153)	(.106)	(.102)
Accountability	0.123			
	(.316)			
Corruption	−0.348			
	(.328)			
Government efficiency	−0.075			
	(.413)			
Political stability	0.057			
	(.143)			
Regulatory quality	0.19			
	(.305)			
Rule of law	−0.187			
	(.336)			
Year	0.009		0.015	
	(.024)		(.020)	
SSA			1.289*	1.342*
			(.340)	(.329)
LAC			1.096*	1.039*
			(.276)	(.269)
Constant	−28.718	−10.30*	−40.498	−10.061*
	(47.722)	(1.165)	(38.911)	(.817)
Best model^1^	FE	FE	RE	RE
Overall R-squared	0.308	0.311	0.528	0.527
Observations	647	647	647	647
Number of countries	125	125	125	125

Robust standard errors in parentheses.

* p < 0.001.

^1^Indicated by the Hausman and Sargan-Hansen Tests.

As an alternative estimation method, we looked at HIV domestic/public spending per person living with HIV. All of the coefficients retained the same sign and significance level except that prevalence became negative.

## Trends in domestic-public spending

**Figure 1:**
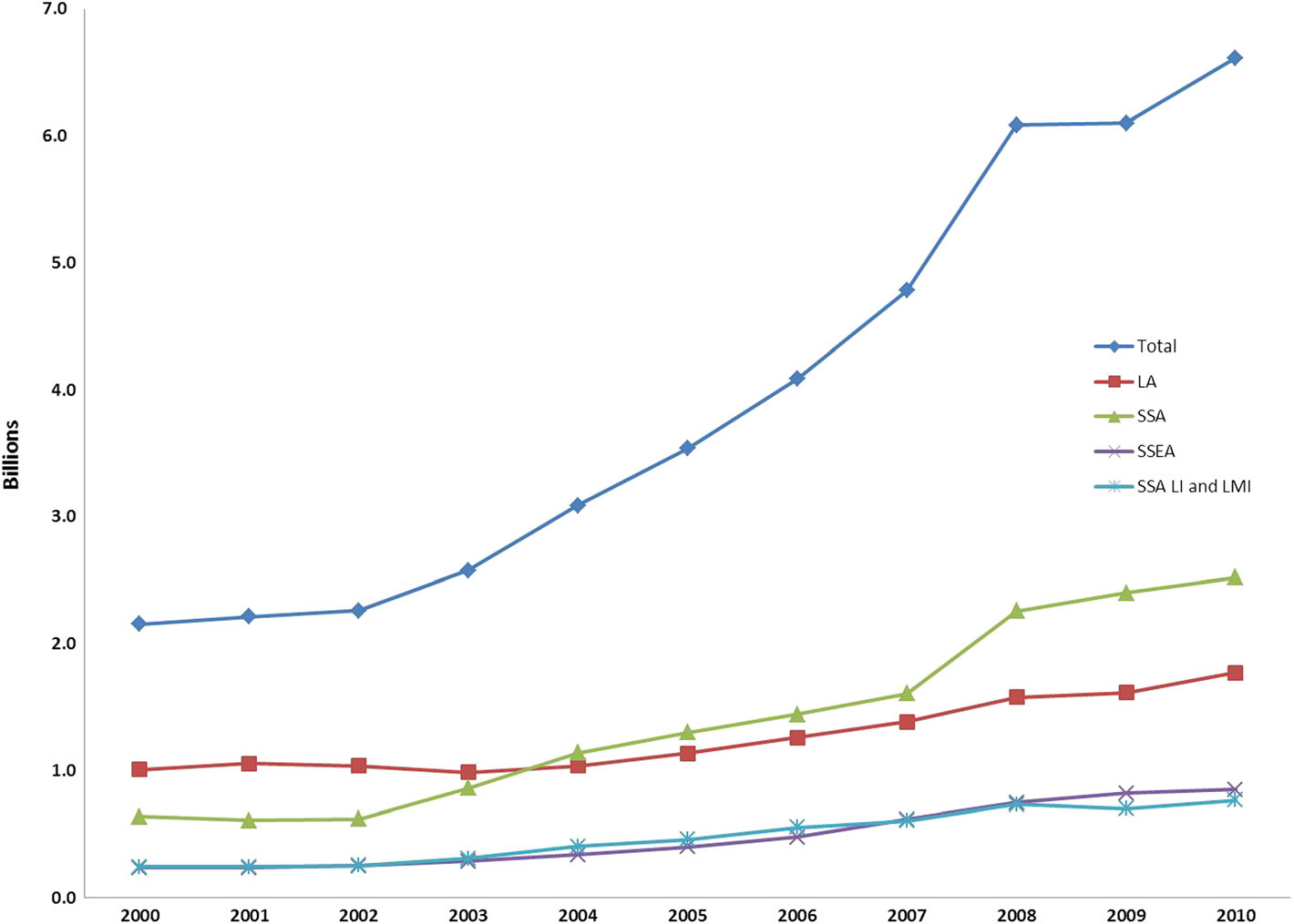
Domestic Public HIV Spending Globally and by Region (2000-2010).

Globally, domestic/public funding of HIV in low- and middle-income countries has increased from US$ 2.15 billion in 2000 to more than US$ 6.6 billion in 2010. This growth rate has mostly been constant, but there was an overall stagnation between 2008 and 2009 as the global economy was shaken. Regionally, SSA overtook LAC in 2004 and has remained the region with the highest amount of government financed resources devoted to HIV. Figure 1 shows the total domestic public spending for HIV by region between 2000 and 2010. The growth in SSA was mainly driven by upper-middle income countries. Of these six countries, Botswana and South Africa are the primary drivers of the growth in HIV spending in SSA. If these upper-middle income countries are excluded, the growth of domestic/public funding in SSA almost mirrors the growth of South and South-East Asia (SSEA).

Overall, per capita HIV spending from domestic sources increased from US$ 0.31 in 2000 to US$ 1.11 in 2010 and varied widely by type of epidemic, income level and region (Table 3). In 2010 spending ranged from 5 cents in low-level epidemics in the Middle East and North Africa to US$ 32 in upper-middle income countries with generalized epidemics in East and Southern Africa. Countries with low-level epidemics spent US$ 0.34 and those with generalized epidemics US$ 3.04 per capita. Low-income countries spent an average of US$ 0.45 per capita and upper-middle-income countries almost US$ 5.00. Regional spending also showed wide variation with per-capita spending from US$ 0.20 in the Middle East and North Africa to US$ 3.45 in sub-Saharan Africa. Additional file 1: Appendix 1 and Additional file 1: Appendix 2 present regional and country domestic public expenditures on HIV based on our full database of 1595 country years from 145 low- and middle-income countries. All the predicted values were substituted by reported country data when available.

**Table 3:** Domestic public spending per-capita in 2010

	Countries income level *			
**Type of HIV epidemic**	**Low-income (53)**	**Lower-middle-income (53)**	**Upper-middle-income (39)**	**ALL (145)**
	**GDP < US$ 935**	**GDP < US$ 936 - 3,705>**	**GDP > US$ 3,706**	
Generalized (39)**	$0.81	$3.38	$31.58	$3.04
Concentrated (47)	$0.39	$0.45	$3.53	$0.92
Low level (59)	$0.05	$0.30	$1.33	$0.34
**Region**				
The Caribbean (12)	$0.05	$2.48	$4.02	$1.83
Eastern Europe and Central Asia (18)	$0.28	$0.72	$4.00	$2.50
Latin America (18)	n/a	$1.42	$3.74	$3.14
Middle East and North Africa (16)	$0.05	$0.25	$1.01	$0.20
Oceania(16)	$0.28	$0.80	$0.43	$0.37
Sub-Saharan Africa (43)	$0.96	$3.41	$30.83	$3.45
South and South-East Asia (20)	$0.32	$0.56	$0.93	$0.37
Western and Central Europe (14)	n/a	$0.42	$1.38	$1.33
ALL (145)	$0.45	$0.48	$4.97	$1.11

*Income level using the World Bank Atlas Method [13].

**Numbers in parenthesis are number of observations.

**Figure 2:**
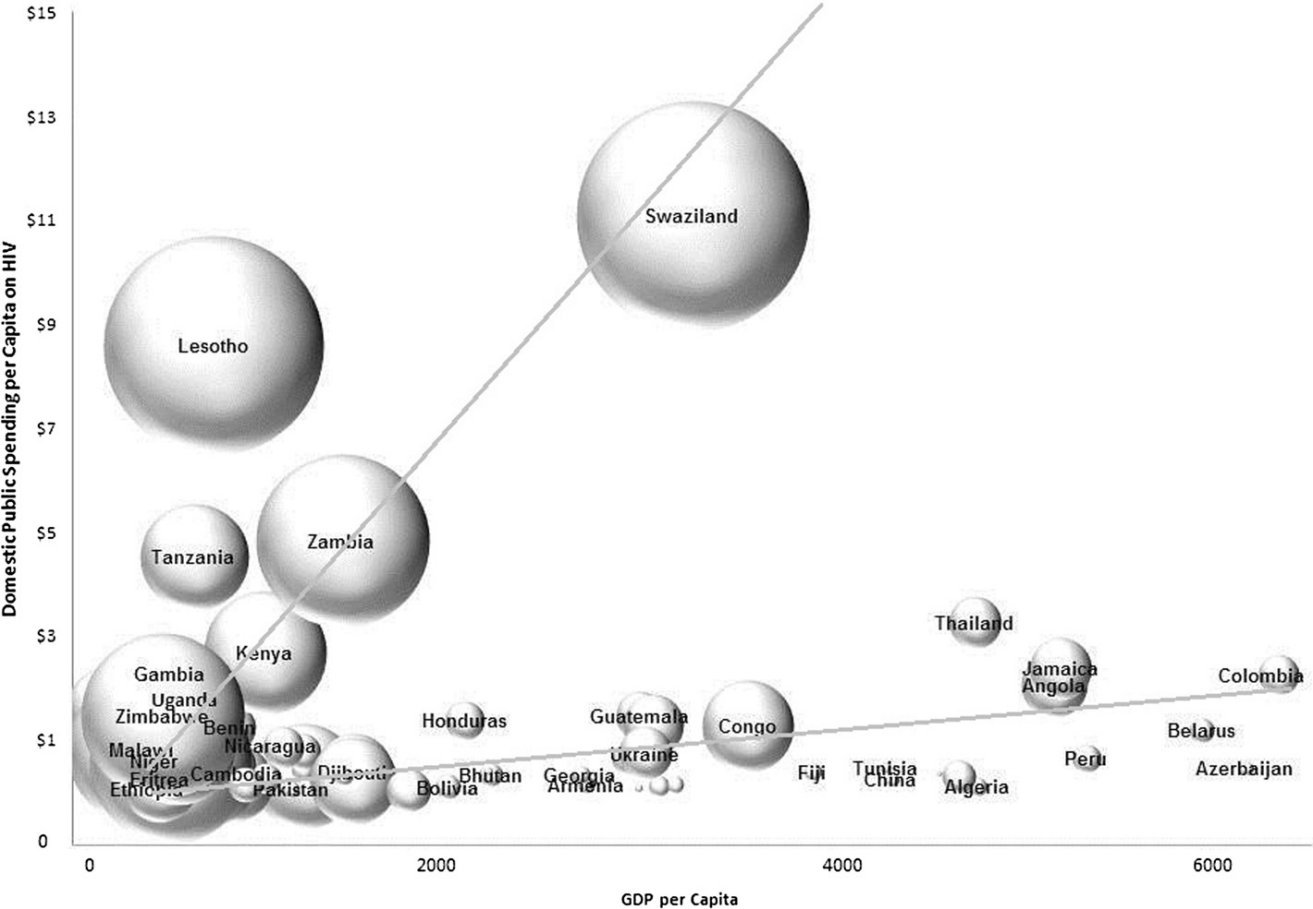
Relationship between GDP and per-capita Spending on AIDS in 2010.

Figure 2 shows the relationship between GDP, on the horizontal axis, the level of domestic per-capita spending on HIV, on the vertical axis, and HIV prevalence, depicted using a spread of bubbles of different sizes. The two regression lines demonstrate the different in trajectory between high and low HIV prevalence countries along with their GDP and domestic health spending. Poor countries with low domestic spending on HIV and high prevalence are located in the lower left quadrant and have large bubbles. These countries are especially vulnerable and more likely unable to invest enough resources to control the spread of HIV due to their low income levels and high rates of HIV prevalence. This is the case for Zimbabwe, Kenya, Uganda and Malawi, which have low domestic spending on HIV, a high HIV burden and low economic capacity.

**Figure 3:**
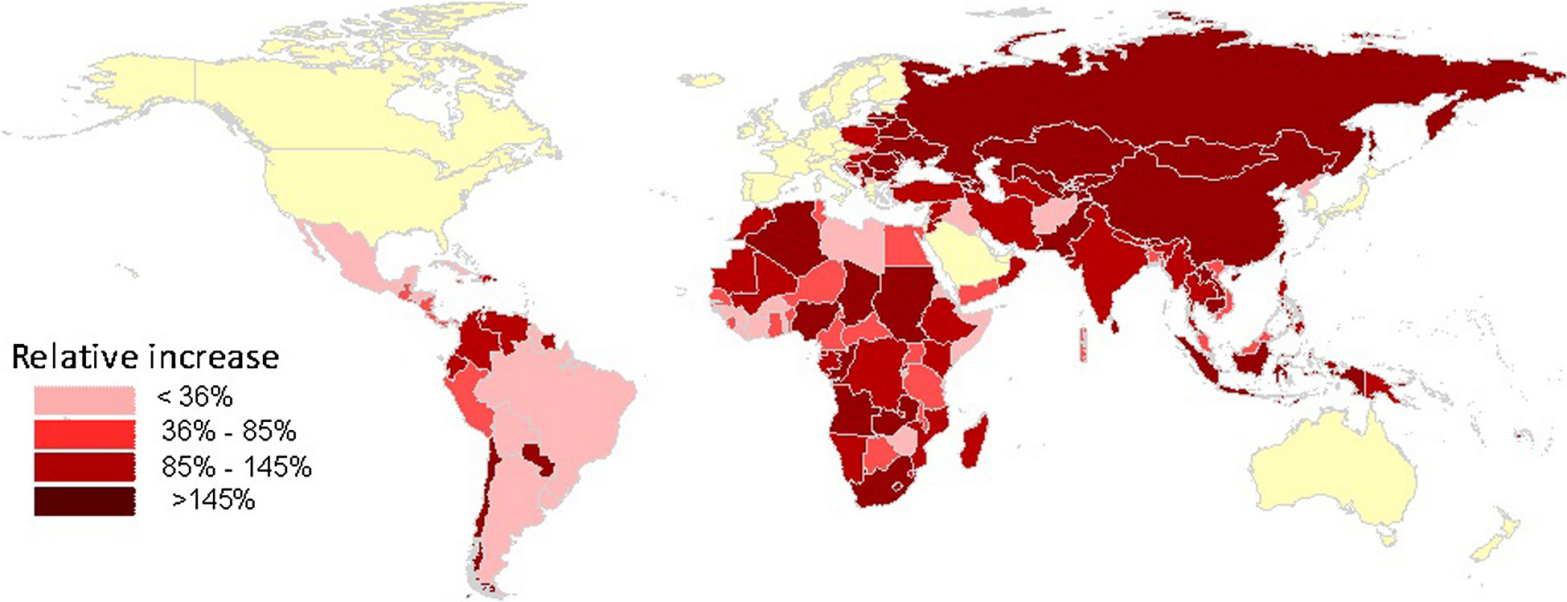
Percentage change in domestic public HIV expenditures during 2005-2009 as compared to 2000-2004.

Figure 3 presents the trends of domestic public spending in all developing countries. There was a substantial increase in government financing for HIV with substantial increases in sub-Saharan Africa, Eastern Europe and Asia. Even in low-income sub-Saharan Africa, aggregated public financing increased from US$ 639 million to US$ 2.5 billion in 2010, an almost four-fold increase. In addition, the amount of resources committed by governments has been increasing and almost matched international aid over the past eleven years.

## Discussion and conclusion

During the last decade, domestic resources have been instrumental in the efforts against HIV. Low- and middle-income countries invested a total of US$ 43.5 billion from domestic public sources in the last decade, reaching US$ 6.6 billion in 2010, a three-fold increase in domestic funding from the US$ 2.15 billion recorded in 2000. The total amount from domestic sources and spent in the 125 countries analysed represents almost one half of the funding available for HIV in these countries during the last decade. In 2010 alone, low- and middle- income countries (LMIC) contributed more than US $ 7.6 billion from public (US$ 6.6 billion) and private sources (US$ 1.0 billion) to their domestic HIV responses, while international funding from bilateral and multilateral organizations and the philanthropic sector contributed a total of US$ 7.5 billion for a combined amount of US$ 15.1 billion in 2010 [6].

This analysis also found that GDP and HIV prevalence are positively associated with the level of domestic spending from public sources. A large body of evidence shows a strong and positive correlation between national income and national expenditures on health care and is a consistent finding of research [7,8]. Many argue that developing countries’ ability to take on a greater share of the financial burden is critical to ensuring long-term sustainable financing and this may be possible with increased economic growth.

In most countries the domestic HIV response is still not commensurate with the magnitude of the epidemic [3]. Approaches that contribute to greater financial sustainability of health programs have been extensively published [9-12]. Governments in low-income countries should mobilize domestic resources and analyse the convenience to implement innovative financing mechanisms such as special levies on currency and financial transactions, selling franchised products and services, mobile phone voluntary solidarity contribution, excise tax on tobacco, alcohol and unhealthy food. Still, some low-income countries cannot afford to fully support patients on treatment from domestic resources alone and will need external support.

There are several opportunities to increase public spending for the HIV response to reach the universal access targets for prevention, treatment and care. First, governments can create fiscal space through fiscal instruments such as external grants, domestic revenue mobilization, deficit financing, reprioritization and raising efficiency of current expenditures. There may be substantial opportunity for improving the efficiency of AIDS services, by providing more services with existing resources. A recent study evaluating efficiency of national HIV programs in 68 low- and middle-income countries found only a moderate efficiency of 49.8% in implementing AIDS programs [14]. Countries would also explore improving their capacity to collect progressive and fair taxes from their citizens. Middle-income countries, especially, have more room to create a sustainable tax base. The World Bank estimates that at least 30 per cent of GDP is needed to sustain a well-functioning state, but some government budgets are below 20 percent of GDP [15].

Middle-income countries have the potential to increase their domestic spending sustainably, especially those less affected by the economic crisis, notably South Africa [16]. A recently proposed HIV domestic priority index shows a country’s ability to fund its own HIV response [17]. Middle-income countries would seem to have potential to increase their domestic spending sustainably. China and India, two countries with increasing epidemics, are spending at relatively low levels given their disease burden and ability to pay. Therefore, they could contribute more to the HIV response from domestic resources. Even one of the weakest economies in Africa, Zimbabwe, has shown unprecedented commitment and has been able to augment the government budgetary allocations to HIV programs by introducing a monthly income tax from employee salaries [18].

This study describes public expenditures from an international perspective and acknowledges that these should provide information on beneficiary populations. It has been shown that in spite of the fact that the HIV epidemic in Latin America is driven by MSM and sex workers, the majority of prevention spending is not targeted at these groups [19]. At the country level, benefit-incidence analyses (BIA) have the potential to complement resource tracking activities by analyzing who benefits from using health services. While traditionally BIA has focused on pro-poor subsidies, the same methodology can be used to assess how well HIV programs are performing in terms of the distribution of service benefits [20].

The economic outlook for low and middle income countries is promising; even in Africa, GDP is expected to grow by nearly 6 percent in 2013. This economic growth is widespread across Africa and more than half of all countries have improved governance with a majority of countries improving in human development and a growing middle class; these shifts suggest a dynamic cycle of domestic growth [21-23].

Sustained increases in domestic funding for HIV were observed in all regions during the last decade. Sub-Saharan Africa has made a formidable effort characterized by higher domestic/public spending in spite of a lower GDP per-capita and higher prevalence than any other region. Despite huge income disparities, sub-Saharan Africa and Latin America devoted significant domestic resources to financing HIV-related activities, the former under the pressure of high demand and a high disease burden, the latter perhaps facilitated by their developed health systems. Lower amounts were spent in the Middle East, North Africa and Southeast Asia relative to their populations, income level and government revenue.

Africa, in particular sub-Saharan Africa, is home to 80 percent of people living with HIV globally and one million people living on less than a dollar a day. They spent 0.22 percent of their GDP, equivalent to US$ 2.5 billion or US$ 3.45 per capita in 2010, the highest per capita spending amongst low- and middle-income countries. This region requires coordinated investments from international donors as well as from national governments to curb the continuous and chronic effects of the HIV epidemic.

The 2011 Political Declaration on HIV recognizes that this worldwide commitment to the global HIV epidemic has been unprecedented and represents the largest amount dedicated to combating a single disease in history [24]. However, international assistance from bilateral organizations declined 10 percent between 2009 and 2010, following years of steady increases in funding which increases the financial burden on low- and middle-income countries in the coming years [25]. Assistance by international organizations such as GF, UNITAID and PEPFAR have been critical, accounting for most of the funding for HIV in many low income and high burden countries and US$ 7.5 billion in 2010 alone. HIV directed foreign aid has significantly positive effects on a country’s treatment coverage rates [26]. In fact, the expansion of access to HIV antiretroviral treatment to 8 million people by the end of 2011 has resulted in the reduction of HIV-related deaths by more than 20 per cent in the past five years.

As in any study, there were several limitations. Domestic spending is reported using government sources of information, which in some countries lack comprehensive expenditure records and the accounting information systems do not contain specific budgetary and expenditure lines related to HIV. On balance, these data issues suggest that under-estimation is more likely than overestimation and our results represent lower bound estimates. Development partners and governments are increasing efforts to strengthen resource tracking over time. The estimation of missing data points may be vulnerable to any unobserved effects that we were not able to capture in the prediction model. In spite of the relatively robust methodology and the use of panel analysis based on a random effects model, there is always the possibility of unobserved variables that were omitted from the analysis. This analysis does not include out-of-pocket expenditures; although out-of-pocket spending has been found to vary from 23 to 68% of total health expenditures, the proportion that households divert to the purchase of condoms, HIV testing, clean syringes or other preventive interventions is unknown [27].

Sustainable domestic investments are needed to achieve a robust response to the HIV epidemic which will yield long-term dividends. The high level of dependence on international funding in low-income countries requires rethinking the global financial aid architecture to safeguard the sustainability of HIV funding [28]. International donors should be aware that the political economy of global HIV finance influences domestic public finance and both domestic and international sources should explore ways to strategically increase their combined and complementary investments. It is important to recognize that HIV is a shared responsibility and ownership is instrumental. Countries should be able to access predictable and sustainable HIV resources aligned with national development strategies that maximize synergies and are implemented with transparency and accountability [29].

## Competing interest

The authors declare that they have no competing interests.

## Authors’ contributions

CA, DL and PDL designed research. DL and PA conducted statistical analysis. PA, PDL and CA wrote the paper and CA had primary responsibility for final content. All authors read and approve the final manuscript.

## Pre-publication history

The pre-publication history for this paper can be accessed here:

http://www.biomedcentral.com/1471-2458/13/673/prepub

## Supplementary Material

Additional file 1 **Appendix 1.** Total domestic public expenditures on HIV by year and region in US$ million. Appendix 2. Domestic public expenditures on HIV in thousand USD, based on our full database of 1595 country years from 145 low- and middle-income countries. Predicted values in Bold. Click here for file

## References

[B1] Haacker M Financing HIV/AIDS programs in sub-Saharan Africa Health Aff 2009 28 6 1606 10.1377/hlthaff.28.6.1606 19887402

[B2] UNAIDS Together We Will End AIDS 2012 Geneva: UNAIDS

[B3] Amico P Aran C Avila C HIV spending as a share of total health expenditure: an analysis of regional variation in a multi-country study PLoS One 2010 5 9 e12997 10.1371/journal.pone.0012997 20885986 PMC2945774

[B4] UNAIDS National AIDS Spending Assessment: A Notebook on Methods Definitions and Procedures to Measure HIV and AIDS Financial Flows and Expenditures at the Country Level 2009 Geneva: UNAIDS

[B5] World Bank The Worldwide Governance Indicator: Methodology and Analytical Issues. In 2010 Washington DC: World Bank

[B6] Kates J Wexler A Lief E Gobet B Financing the Response to AIDS in Low- and Middle- Income Countries: International Assistance from Donor Governments in 2011 2012 Kaiser Family Foundation and UNAIDS available at: http://www.unaids.org/en/media/unaids/contentassets/documents/document/2012/201207_KFF-UNAIDS-2012-Report_en.pdf. Accessed 31 July 2013

[B7] Hansen P King A The determinants of health care expenditure: A cointegration approach J Health Econ 1996 15 1 127 137 10.1016/0167-6296(95)00017-8 10157426

[B8] Youde J The relationships between foreign aid, HIV and government health spending Health Policy Plan 2010 25 6 523 528 10.1093/heapol/czq030 20663811

[B9] Routh S Thwin AA Baqui AH Cost-effectivenes and Sustainability Aspects of MCH-FP Programmes in Bangladesh. ICDDR, B Working Paper No. 100 1997 Dhaka: International Centre for Diarrhoeal Disease Research

[B10] Rao P Gabre-Kidan T Mubangizi DB Sulzbach S Leveraging the private health sector to enhance HIV service delivery in lower-income countries Journal of Acquired Immune Deficiency Syndromes 2011 57 Suppl. 2 S116 S119 21857294 10.1097/QAI.0b013e31821ed719

[B11] Fryatt R Mills A Taskforce on innovative international financing for health systems: showing the way forward Bull World Health Organ 2010 88 476 477 10.2471/BLT.09.075507 20539869 PMC2878154

[B12] Chris A Fleisher LK Health financing in Africa today: challenges and opportunities 2008 Bethesda, MD: Health Systems 20/20 project, Abt Associates Inc

[B13] World Bank Gross National Income per capita 2009, Atlas Method 2010 Washington: World Bank

[B14] Zeng W Shepard DS Chilingerian J Avila-Figueroa C How much can we gain from improved efficiency? An examination of performance of national HIV/AIDS programs and its determinants in low-and middle-income countries BMC Health Serv Res 2012 12 1 74 10.1186/1472-6963-12-74 22443135 PMC3353196

[B15] Van Damme W World social health insurance: Strengthening health systems in low-income countries PLoS Med 2007 4 3 e137 10.1371/journal.pmed.0040137 17388675 PMC1831747

[B16] IMF World Economic Outlook 2010 Washington: IMF

[B17] UNAIDS 2010 Global Report: UNAIDS Global Report on the AIDS Epidemic 2010 Geneva: UNAIDS

[B18] UNAIDS 2012 Zimbabwe: AIDS levy generates new resources for treatment Retrieved 1 July, 2012, from http://www.unaids.org/en/resources/presscentre/featurestories/2012/february/20120221zimbabwe/

[B19] Arán-Matero D Amico P Arán-Fernandez C Gobet B Izazola-Licea JA Avila-Figueroa C Levels of Spending and Resource Allocation to HIV Programs and Services in Latin America and the Caribbean PLoS One 2011 6 7 e22373 10.1371/journal.pone.0022373 21799839 PMC3142155

[B20] McIntyre D Ataguba JE How to do (or not to do)… a benefit incidence analysis Health Policy Plan 2011 26 2 174 182 10.1093/heapol/czq031 20688764

[B21] IMF World Economic Outlook 2011 Washington DC: IMF

[B22] 2011 Ibrahim Index of African Governance 2011 Published October 2012. Mo Ibrahim Foundation

[B23] African Development Bank African Economic Outlook 2011 2011 African Development Bank, Organisation for Economic Co-operation and Development, United Nations Development Programme, United Nations Economic Commission for Africa (2011). OECD Publishing Available at: http://www.undp.org/content/dam/undp/library/corporate/Reports/UNDP-Africa-2011-Economic-Outlook.pdf. Accessed 31 July 2013

[B24] UN General Assembly Political Declaration on HIV/AIDS 2011 New York: UN

[B25] Kates J Boortz K Lief E Avila C Gobet B Financing the Response to AIDS in Low- and Middle- Income Countries; International Assistance from the G8, European Commission and Other Donor Governments in 2009 2010 Menlo Park: Kaiser Family Foundation & UNAIDS

[B26] Peiffer CA Boussalis C Foreign assistance and the struggle against HIV/AIDS in the developing world J Dev Stud 2010 46 3 556 573 10.1080/00220380903151041

[B27] Leive A Xu K Coping with out-of-pocket health payments: empirical evidence from 15 African countries Bull World Health Organ 2008 86 11 849 856C 10.2471/BLT.07.049403 19030690 PMC2649544

[B28] 4th High Level Forum on Aid Effectiveness Busan Partnership For Effective Development Co-operation 2011 Busan, Korea: OECD

[B29] Ghebreyesus TA Achieving the health MDGs: country ownership in four steps Lancet 2010 376 9747 1127 1128 10.1016/S0140-6736(10)61465-1 20864154

