# Supplementary material for: Determinants of government HIV/AIDS financing: a 10-year trend analysis from 125 low- and middle-income countries
Source: BMC Public Health. 2013 Jul 19;13:673. doi: 10.1186/1471-2458-13-673 (PMC3733926; doi:10.1186/1471-2458-13-673)
Supplement: Additional file 1 — Appendix 1. Total domestic public expenditures on HIV by year and region in US$ million. Appendix 2. Domestic public expenditures on HIV in thousand USD, based on our full database of 1595 country years from 145 low- and middle-income countries. Predicted values in Bold. [file 1471-2458-13-673-S1.docx]

**Appendix 1. Total domestic public expenditures on HIV by year and region in US$ million**

| **Region** | **2000** | **2001** | **2002** | **2003** | **2004** | **2005** | **2006** | **2007** | **2008** | **2009** | **2010** | **Grand Total** |
| --- | --- | --- | --- | --- | --- | --- | --- | --- | --- | --- | --- | --- |
| Caribbean | 46 | 48 | 49 | 50 | 51 | 57 | 61 | 74 | 67 | 83 | 69 | 655 |
| East Asia | 46 | 54 | 61 | 71 | 100 | 104 | 128 | 168 | 239 | 272 | 337 | 1,580 |
| Eastern Europe and Central Asia | 77 | 103 | 127 | 169 | 246 | 332 | 448 | 617 | 815 | 582 | 717 | 4,233 |
| Latin America | 1,006 | 1,054 | 1,038 | 986 | 1,037 | 1,137 | 1,261 | 1,386 | 1,580 | 1,614 | 1,772 | 13,870 |
| Middle East and North Africa | 36 | 39 | 41 | 45 | 50 | 57 | 66 | 77 | 92 | 87 | 96 | 686 |
| Oceania | 1 | 1 | 1 | 2 | 2 | 2 | 2 | 3 | 3 | 3 | 3 | 23 |
| Sub-Saharan Africa | 639 | 611 | 619 | 863 | 1,139 | 1,301 | 1,441 | 1,607 | 2,258 | 2,402 | 2,520 | 15,401 |
| South and South-East Asia | 236 | 236 | 251 | 289 | 338 | 399 | 479 | 616 | 749 | 823 | 851 | 5,269 |
| Western and Central Europe | 68 | 68 | 77 | 101 | 124 | 149 | 197 | 233 | 281 | 237 | 245 | 1,779 |
| **Grand Total** | **2,157** | **2,214** | **2,263** | **2,575** | **3,087** | **3,538** | **4,085** | **4,782** | **6,084** | **6,102** | **6,610** | **43,495** |

**Appendix 2. Domestic public expenditures on HIV in thousand USD, based on our full database of 1595 country years from 145 low- and middle-income countries. Predicted values in Bold.**

| # | Country | 2000 | 2001 | 2002 | 2003 | 2004 | 2005 | 2006 | 2007 | 2008 | 2009 | 2010 |
| --- | --- | --- | --- | --- | --- | --- | --- | --- | --- | --- | --- | --- |
| 1 | Afghanistan | *96* | *100* | *105* | *109* | *113* | *118* | *122* | *126* | ***129*** | ***132*** | *136* |
| 2 | Albania | *441* | *500* | *552* | *743* | *980* | ***1,110*** | *1,242* | *1,513* | *1,891* | *1,757* | *1,717* |
| 3 | Algeria | *869* | *907* | *981* | *1,244* | *1,667* | ***6,740*** | ***2,496*** | ***2,976*** | ***4,051*** | ***3,319*** | *3,821* |
| 4 | Angola | *3,021* | *2,933* | *3,869* | *4,888* | ***8,897*** | ***18,311*** | ***27,724*** | ***39,012*** | ***39,171*** | ***30,730*** | *39,470* |
| 5 | Argentina | ***191,672*** | ***181,472*** | ***152,252*** | ***79,695*** | ***97,020*** | ***119,705*** | ***144,589*** | ***203,970*** | ***242,290*** | ***243,745*** | ***249,346*** |
| 6 | Armenia | *79* | ***88*** | ***100*** | ***120*** | ***196*** | ***271*** | ***336*** | ***469*** | ***636*** | ***633*** | *424* |
| 7 | Azerbaijan | *205* | *231* | *263* | *325* | *413* | *698* | *1,238* | ***2,180*** | ***3,364*** | ***4,145*** | *4,211* |
| 8 | Bangladesh | *1,478* | *1,573* | *1,743* | *2,021* | *2,344* | *2,546* | *2,856* | *3,454* | *4,193* | *4,967* | *5,831* |
| 9 | Belarus | *1,725* | ***2,179*** | ***2,740*** | ***4,117*** | ***5,815*** | ***6,598*** | ***8,955*** | ***10,956*** | ***14,033*** | ***11,352*** | *11,627* |
| 10 | Belize | *577* | ***611*** | ***659*** | ***1,143*** | ***1,061*** | ***979*** | ***898*** | ***903*** | ***959*** | ***929*** | *950* |
| 11 | Benin | *3,399* | *3,598* | *4,060* | *5,263* | ***10,595*** | ***9,030*** | ***7,465*** | ***8,470*** | ***10,492*** | ***13,018*** | *11,660* |
| 12 | Bhutan | *33* | *39* | *47* | *57* | *71* | *90* | *105* | *159* | *174* | *197* | *231* |
| 13 | Bolivia | ***439*** | ***778*** | ***922*** | ***673*** | ***454*** | ***496*** | ***609*** | ***711*** | ***945*** | ***969*** | *1,053* |
| 14 | Bosnia_and_Herzegovina | *82* | *86* | *97* | *127* | *155* | *168* | *195* | *251* | ***313*** | ***300*** | *289* |
| 15 | Botswana | *75,741* | ***82,248*** | ***93,611*** | ***117,421*** | ***145,064*** | ***165,043*** | ***166,104*** | ***203,796*** | ***205,362*** | *170,379* | *191,955* |
| 16 | Brazil | ***498,513*** | ***509,200*** | ***519,886*** | ***530,573*** | ***541,260*** | ***551,946*** | ***562,633*** | ***572,306*** | ***616,881*** | ***725,940*** | ***835,000*** |
| 17 | Bulgaria | *890* | *1,044* | *1,268* | *1,744* | *2,287* | ***2,753*** | ***3,224*** | ***4,337*** | ***6,076*** | ***5,639*** | *5,825* |
| 18 | Burkina_Faso | *3,324* | ***3,522*** | ***5,383*** | ***9,340*** | ***11,004*** | ***8,013*** | ***9,651*** | ***8,558*** | ***10,702*** | *10,397* | *11,229* |
| 19 | Burundi | *8,768* | *7,783* | ***7,157*** | ***6,650*** | ***18,619*** | ***13,964*** | ***11,054*** | ***11,907*** | ***14,715*** | *17,138* | *18,812* |
| 20 | Cambodia | *1,735* | ***1,870*** | ***1,977*** | ***2,115*** | ***2,406*** | ***3,534*** | ***6,055*** | ***6,045*** | ***5,734*** | *5,372* | *5,663* |
| 21 | Cameroon | *2,624* | *2,444* | *2,793* | *3,581* | ***4,197*** | ***4,411*** | ***6,002*** | ***6,839*** | ***6,732*** | *5,942* | *6,188* |
| 22 | Cape_Verde | *197* | *207* | *231* | *315* | *363* | *398* | ***800*** | ***1,791*** | ***753*** | ***760*** | *802* |
| 23 | Central_African_Republic | *405* | *410* | *436* | ***639*** | ***691*** | ***743*** | ***652*** | ***760*** | ***903*** | *892* | *958* |
| 24 | Chad | *296* | *381* | *459* | ***618*** | ***1,074*** | ***1,491*** | ***1,943*** | ***2,940*** | ***2,243*** | *1,731* | *2,038* |
| 25 | Chile | ***24,112*** | ***21,872*** | ***21,492*** | ***24,061*** | ***32,375*** | ***45,193*** | ***59,173*** | ***73,154*** | ***87,134*** | *59,223* | *73,659* |
| 26 | China | *44,841* | *51,996* | *59,375* | ***69,635*** | ***97,992*** | ***102,052*** | ***125,540*** | ***165,650*** | ***236,039*** | ***268,775*** | ***334,328*** |
| 27 | Colombia | ***39,096*** | ***35,943*** | ***36,014*** | ***51,156*** | ***66,298*** | ***81,440*** | ***96,582*** | ***68,614*** | ***103,056*** | ***108,241*** | ***108,241*** |
| 28 | Comoros | *49* | *55* | *66* | *90* | *105* | *116* | *124* | ***150*** | *179* | *184* | *201* |
| 29 | Congo | *1,137* | *949* | *1,022* | ***1,196*** | ***1,637*** | ***4,719*** | ***2,863*** | ***3,091*** | ***4,566*** | ***9,132*** | *4,879* |
| 30 | Costa_Rica | ***8,997*** | ***8,065*** | ***8,130*** | ***8,426*** | ***9,013*** | ***9,755*** | ***11,129*** | ***14,307*** | ***18,521*** | *14,627* | *16,462* |
| 31 | Cote_d_Ivoire | *3,004* | *3,003* | *3,278* | ***3,963*** | ***5,248*** | ***4,451*** | ***4,705*** | ***6,127*** | ***6,551*** | *5,624* | *5,769* |
| 32 | Croatia | *2,668* | *2,884* | *3,408* | *4,524* | *5,591* | *6,184* | ***6,930*** | ***8,826*** | ***10,317*** | ***10,177*** | *9,358* |
| 33 | Cuba | *34,510* | *35,863* | *37,161* | *38,356* | *39,441* | *40,394* | *42,776* | ***44,543*** | ***45,191*** | ***62,670*** | *46,388* |
| 34 | DR_Congo | *1,097* | *1,350* | *1,469* | *1,510* | *1,780* | ***3,622*** | *2,478* | ***2,860*** | ***3,388*** | *3,212* | *3,713* |
| 35 | DR_Korea | *1,499* | *1,525* | *1,539* | *1,553* | *1,566* | *1,578* | *1,589* | *1,600* | *1,611* | *1,621* | *1,631* |
| 36 | Djibouti | *156* | *164* | *170* | *180* | *194* | *207* | *225* | *250* | *293* | *312* | *336* |
| 37 | Dominica | *22* | *22* | *21* | *21* | *23* | *25* | *26* | *29* | ***31*** | ***31*** | *32* |
| 38 | Dominican_Republic | *4,786* | *4,985* | *4,437* | *3,944* | *4,240* | ***7,009*** | ***8,311*** | ***16,602*** | ***9,899*** | *10,198* | *11,039* |
| 39 | Ecuador | *953* | *1,395* | *1,604* | ***1,860*** | ***2,500*** | ***2,625*** | ***3,255*** | ***3,202*** | *3,692* | *3,884* | *4,240* |
| 40 | Egypt | *1,929* | *1,903* | *1,774* | *1,680* | *1,662* | *1,979* | *2,492* | ***3,426*** | ***4,091*** | *4,908* | *5,817* |
| 41 | El_Salvador | ***15,441*** | ***16,737*** | ***17,735*** | ***22,265*** | ***23,705*** | ***25,438*** | ***27,171*** | ***34,757*** | ***31,274*** | *28,891* | *30,010* |
| 42 | Equatorial_Guinea | *45* | *67* | *89* | *133* | *267* | *461* | *566* | ***969*** | ***1,251*** | ***919*** | *1,070* |
| 43 | Eritrea | *472* | *496* | *465* | *555* | *713* | ***687*** | ***728*** | ***786*** | ***846*** | ***1,217*** | *1,386* |
| 44 | Ethiopia | *1,470* | *1,439* | *1,318* | *1,407* | *1,774* | *2,176* | *2,686* | *3,494* | *4,859* | *5,896* | *5,424* |
| 45 | Fiji | *119* | *120* | *139* | ***185*** | ***229*** | ***288*** | ***330*** | ***371*** | ***398*** | ***295*** | *323* |
| 46 | Gabon | *3,323* | *3,045* | *3,235* | *4,058* | *4,872* | ***6,709*** | ***6,617*** | ***8,139*** | ***10,588*** | ***9,454*** | *9,607* |
| 47 | Gambia | *1,296* | *1,333* | *1,218* | *1,251* | ***5,543*** | ***3,770*** | ***2,017*** | ***2,777*** | ***3,742*** | *3,410* | *3,922* |
| 48 | Georgia | *238* | *263* | *292* | ***411*** | ***503*** | ***668*** | ***838*** | ***1,183*** | ***1,588*** | ***2,233*** | *1,390* |
| 49 | Ghana | *3,378* | *3,604* | ***4,225*** | ***9,268*** | ***8,559*** | ***7,850*** | ***10,174*** | ***11,248*** | ***11,820*** | *10,677* | *12,337* |
| 50 | Grenada | *196* | *193* | *200* | *223* | *217* | *262* | *268* | ***293*** | ***484*** | ***296*** | *312* |
| 51 | Guatemala | ***10,230*** | ***9,664*** | ***9,992*** | ***11,187*** | ***13,879*** | ***13,978*** | ***15,852*** | *18,325* | *21,576* | *20,535* | *22,162* |
| 52 | Guinea | *220* | *214* | *228* | ***245*** | ***260*** | ***271*** | ***195*** | ***291*** | ***317*** | ***665*** | *295* |
| 53 | Guinea_Bissau | *329* | ***516*** | ***516*** | ***516*** | ***516*** | ***402*** | *448* | *480* | ***564*** | ***653*** | *750* |
| 54 | Guyana | ***557*** | ***567*** | ***716*** | ***841*** | *629* | *660* | *738* | *894* | *981* | *1,023* | *1,092* |
| 55 | Haiti | *270* | *238* | ***225*** | ***185*** | ***225*** | ***331*** | ***439*** | *394* | *449* | *447* | *439* |
| 56 | Honduras | ***6,141*** | ***6,609*** | ***6,510*** | ***6,411*** | ***6,313*** | ***6,654*** | ***7,589*** | ***8,812*** | ***10,121*** | *10,409* | *11,264* |
| 57 | Hungary | *1,084* | *1,256* | *1,638* | *2,143* | *2,712* | *2,946* | *3,033* | ***4,550*** | ***4,362*** | ***3,535*** | *4,064* |
| 58 | India | *137,449* | ***139,865*** | ***145,235*** | ***168,908*** | ***197,985*** | ***232,652*** | ***279,052*** | ***381,928*** | ***484,804*** | ***529,194*** | ***545,070*** |
| 59 | Indonesia | *2,461* | ***2,950*** | ***4,258*** | ***6,406*** | ***9,550*** | ***13,000*** | ***15,038*** | ***17,744*** | ***22,270*** | *24,365* | *32,078* |
| 60 | Iran | *8,038* | ***10,320*** | ***10,694*** | ***12,594*** | ***15,443*** | ***21,875*** | ***29,750*** | ***30,649*** | ***33,327*** | *32,755* | *35,945* |
| 61 | Iraq | *10,756* | *11,079* | *11,411* | *11,753* | *12,106* | *5,542* | *8,366* | *10,907* | *17,556* | *12,773* | *15,983* |
| 62 | Jamaica | *4,550* | *4,605* | *4,942* | ***4,770*** | ***5,193*** | ***6,160*** | ***6,831*** | ***9,215*** | *7,525* | *6,251* | *7,018* |
| 63 | Jordan | *258* | *276* | *296* | *317* | *359* | *402* | *483* | ***563*** | ***723*** | ***1,245*** | *863* |
| 64 | Kazakstan | *998* | *1,364* | *1,681* | *2,360* | *3,719* | *5,514* | *8,593* | ***12,578*** | ***17,145*** | ***16,267*** | *17,178* |
| 65 | Kenya | *24,137* | *26,424* | ***27,413*** | ***33,073*** | ***35,836*** | ***43,332*** | ***54,325*** | ***68,198*** | ***77,983*** | ***97,765*** | ***108,600*** |
| 66 | Kyrgyzstan | *265* | *312* | *344* | *435* | ***525*** | ***607*** | ***729*** | ***1,073*** | ***1,487*** | ***1,813*** | *1,518* |
| 67 | Lao_DR | *22* | ***25*** | ***25*** | ***28*** | ***34*** | ***39*** | ***53*** | ***68*** | ***99*** | ***115*** | *99* |
| 68 | Latvia | *727* | *812* | *964* | *1,228* | *1,596* | ***1,939*** | ***5,674*** | ***4,495*** | ***4,736*** | ***3,519*** | *3,168* |
| 69 | Lebanon | *1,358* | *1,449* | *1,653* | *1,816* | *2,040* | *2,153* | *2,275* | ***3,200*** | ***3,212*** | ***3,744*** | *4,188* |
| 70 | Lesotho | *7,661* | *6,902* | *6,251* | *9,702* | ***12,561*** | ***13,678*** | ***14,687*** | ***20,007*** | ***46,293*** | *16,402* | *17,914* |
| 71 | Liberia | *124* | *120* | *127* | *92* | ***105*** | *121* | *140* | *169* | *216* | *214* | *225* |
| 72 | Libyan_Arab_Jamahiriya | *7,496* | *7,646* | *7,797* | *7,952* | *8,109* | *8,270* | *8,434* | *8,602* | *8,773* | *8,947* | *9,125* |
| 73 | Lithuania | *759* | ***838*** | *1,021* | *1,424* | *1,804* | *2,158* | *2,586* | *3,513* | *4,366* | *3,338* | *3,129* |
| 74 | Macedonia_FYR | *1,841* | *1,847* | *1,852* | *1,856* | *1,860* | ***1,866*** | ***1,872*** | ***1,938*** | ***2,066*** | *1,888* | *1,894* |
| 75 | Madagascar | *1,099* | *1,301* | *1,243* | ***1,578*** | ***1,199*** | ***1,395*** | ***1,623*** | ***3,060*** | ***5,410*** | *2,433* | *2,352* |
| 76 | Malawi | *3,907* | ***3,835*** | ***6,191*** | ***5,525*** | ***10,680*** | ***18,264*** | ***12,808*** | ***8,100*** | ***9,861*** | ***11,834*** | *12,559* |
| 77 | Malaysia | *9,696* | *9,569* | *10,619* | *11,889* | *13,856* | *15,692* | *18,371* | *22,548* | ***27,709*** | ***27,253*** | *26,720* |
| 78 | Maldives | *51* | *47* | *50* | *53* | *58* | *54* | *66* | *78* | *96* | *105* | *115* |
| 79 | Mali | *2,240* | *2,508* | *2,751* | *3,708* | ***4,144*** | ***6,079*** | ***8,659*** | ***5,880*** | ***7,288*** | *7,347* | *7,760* |
| 80 | Marshall_Islands | *108* | *109* | *111* | *113* | *115* | *117* | *120* | ***123*** | *125* | *128* | *131* |
| 81 | Mauritania | *701* | *733* | *753* | *853* | *1,008* | *1,284* | *1,955* | *2,037* | *2,617* | *2,165* | *2,580* |
| 82 | Mauritius | *351* | *359* | *391* | *490* | ***580*** | ***595*** | ***1,061*** | *747* | *970* | *917* | *1,059* |
| 83 | Mexico | *139,736* | ***183,899*** | ***196,833*** | ***189,423*** | ***182,013*** | ***204,368*** | ***235,613*** | ***259,668*** | ***282,010*** | ***221,313*** | *259,002* |
| 84 | Micronesia | *32* | *32* | *32* | *33* | *33* | *33* | *33* | *33* | *33* | *33* | *33* |
| 85 | Moldova | *487* | *607* | *723* | ***930*** | ***1,336*** | ***1,615*** | ***1,896*** | ***2,538*** | ***4,965*** | ***5,547*** | *2,986* |
| 86 | Mongolia | *116* | *131* | *151* | *184* | *249* | ***345*** | ***517*** | ***694*** | ***1,572*** | ***1,157*** | *1,167* |
| 87 | Montenegro | *766* | *762* | *753* | *742* | *732* | *724* | *720* | *720* | *721* | *723* | *726* |
| 88 | Morocco | *1,335* | *1,395* | *1,542* | ***2,005*** | ***2,386*** | ***2,561*** | ***2,919*** | ***5,186*** | ***5,579*** | *4,495* | *4,785* |
| 89 | Mozambique | *4,249* | *4,207* | *4,426* | *5,116* | ***7,326*** | ***11,533*** | ***14,302*** | ***9,652*** | ***12,144*** | *12,035* | *12,562* |
| 90 | Myanmar | *478* | *336* | *341* | *553* | *551* | *626* | *766* | ***1,163*** | ***1,588*** | *1,518* | *1,558* |
| 91 | Namibia | *27,960* | *25,831* | *24,489* | ***37,964*** | ***52,913*** | ***58,226*** | ***64,146*** | ***70,783*** | ***105,650*** | ***95,760*** | *85,256* |
| 92 | Nepal | *148* | *153* | *158* | ***166*** | ***195*** | ***225*** | ***254*** | ***618*** | *377* | *380* | *458* |
| 93 | Nicaragua | ***3,460*** | ***3,589*** | ***3,480*** | ***3,514*** | ***3,834*** | ***4,203*** | ***4,919*** | ***5,748*** | ***6,311*** | *5,407* | *5,505* |
| 94 | Niger | *2,421* | *2,650* | *3,060* | ***6,427*** | ***6,523*** | ***8,932*** | ***11,342*** | ***6,666*** | ***8,567*** | *8,236* | *9,245* |
| 95 | Nigeria | *6,688* | *6,240* | *8,630* | *9,967* | *13,318* | *17,478* | ***23,316*** | ***43,854*** | ***34,461*** | *27,920* | *35,310* |
| 96 | Oman | *171* | *175* | *188* | *211* | *254* | *337* | *417* | *467* | *704* | ***621*** | *748* |
| 97 | Pakistan | *1,976* | ***2,923*** | ***2,409*** | ***2,668*** | ***3,382*** | ***4,028*** | ***5,007*** | ***5,965*** | ***9,718*** | ***15,682*** | *8,608* |
| 98 | Palau | *7* | *7* | *7* | *7* | *7* | *7* | *7* | *7* | *7* | *7* | *7* |
| 99 | Panama | ***8,565*** | ***12,516*** | ***9,957*** | ***9,730*** | ***11,078*** | ***12,427*** | ***13,775*** | ***14,809*** | ***17,648*** | *18,930* | *20,706* |
| 100 | Papua_New_Guinea | *575* | *525* | *544* | *706* | *820* | *987* | *1,147* | *1,342* | *1,752* | *1,727* | *1,952* |
| 101 | Paraguay | ***1,674*** | ***1,398*** | ***1,071*** | ***1,182*** | ***1,525*** | ***1,666*** | ***2,124*** | ***2,908*** | ***6,071*** | ***7,721*** | *4,323* |
| 102 | Peru | ***9,829*** | ***7,267*** | ***6,984*** | ***7,563*** | ***8,689*** | ***10,034*** | ***13,301*** | ***13,950*** | ***18,589*** | ***23,898*** | *19,741* |
| 103 | Philippines | *777* | ***728*** | ***802*** | ***844*** | ***941*** | ***1,294*** | ***2,663*** | ***1,736*** | ***2,084*** | ***2,008*** | *2,323* |
| 104 | Poland | *16,409* | *18,737* | *19,710* | *21,943* | *26,308* | *32,598* | *37,423* | ***48,266*** | ***62,586*** | ***55,520*** | *55,504* |
| 105 | Romania | *14,201* | *15,783* | *18,315* | ***29,723*** | ***36,179*** | ***44,261*** | ***70,850*** | ***81,968*** | ***100,286*** | ***80,101*** | *79,465* |
| 106 | Russian | *64,865* | *87,498* | *108,999* | *145,978* | ***213,851*** | ***288,821*** | ***389,273*** | ***529,038*** | ***700,862*** | *495,652* | *627,858* |
| 107 | Rwanda | *1,533* | *1,464* | *1,451* | ***1,616*** | ***2,733*** | ***2,365*** | ***4,326*** | ***3,609*** | ***4,684*** | *5,341* | *5,880* |
| 108 | Saint_Kitts_Nevis | *720* | *752* | *773* | *799* | *893* | *992* | *1,116* | ***1,343*** | ***1,372*** | ***1,288*** | *1,304* |
| 109 | Saint_Lucia | *138* | *133* | *137* | *147* | *157* | *176* | ***214*** | ***193*** | *202* | *196* | *202* |
| 110 | Saint_Vincent_Grenadines | *109* | *113* | *121* | *127* | *141* | *152* | *175* | *196* | ***207*** | ***221*** | *206* |
| 111 | Samoa | *208* | *220* | *267* | *342* | *397* | *433* | *490* | *575* | *556* | ***619*** | *597* |
| 112 | Sao Tome_Principe | *17* | *17* | *21* | *23* | *25* | *27* | *30* | ***47*** | ***44*** | ***49*** | *52* |
| 113 | Senegal | *3,073* | *3,276* | *3,692* | ***5,943*** | ***11,921*** | ***8,855*** | ***7,190*** | ***8,920*** | ***10,769*** | *10,206* | *10,858* |
| 114 | Serbia | *1,784* | *2,469* | *3,401* | *4,617* | ***5,725*** | ***6,300*** | *7,408* | *10,610* | *13,289* | *11,254* | *11,389* |
| 115 | Seychelles | *380* | *385* | *440* | *446* | *438* | *459* | *509* | ***541*** | ***481*** | ***462*** | *515* |
| 116 | Sierra_Leone | *55* | *73* | *89* | *98* | *112* | *131* | ***154*** | ***201*** | *219* | *208* | *209* |
| 117 | Slovakia | *6,262* | *6,234* | *6,234* | *6,235* | *6,241* | *6,246* | *6,252* | *6,260* | *6,272* | *6,279* | *6,285* |
| 118 | Solomon_Islands | *63* | *61* | *49* | *49* | *56* | *62* | *69* | *92* | ***102*** | ***108*** | *106* |
| 119 | Somalia | *8* | *9* | *9* | *10* | *10* | *11* | *12* | *12* | ***13*** | *14* | *15* |
| 120 | South_Africa | ***316,804*** | ***282,234*** | ***265,234*** | ***428,772*** | ***583,538*** | ***668,968*** | ***711,959*** | ***788,877*** | ***1,305,000*** | ***1,518,000*** | ***1,548,360*** |
| 121 | Sri_Lanka | *591* | *583* | *634* | *731* | *835* | ***2,950*** | ***2,322*** | ***1,695*** | ***1,977*** | ***2,129*** | *1,912* |
| 122 | Sudan | *307* | *340* | *388* | *472* | *591* | *771* | *1,063* | ***1,400*** | *1,791* | *1,660* | *2,036* |
| 123 | Suriname | *166* | *146* | *202* | *242* | *288* | *358* | *434* | ***500*** | *647* | *618* | *677* |
| 124 | Swaziland | *5,246* | *4,569* | *4,131* | ***6,810*** | ***8,922*** | ***10,074*** | ***19,559*** | ***19,559*** | *11,944* | *12,792* | *13,622* |
| 125 | Syria | *609* | *648* | *708* | *664* | *784* | *911* | *1,089* | ***1,499*** | ***1,891*** | ***1,806*** | *2,099* |
| 126 | Tajikistan | *126* | *139* | *166* | *226* | *321* | ***370*** | ***473*** | ***663*** | ***1,016*** | ***1,151*** | *1,101* |
| 127 | Tanzania | *76,624* | *77,344* | *80,860* | *87,422* | *96,890* | ***108,120*** | *109,504* | *129,891* | *165,327* | *180,820* | *201,418* |
| 128 | Thailand | *77,004* | ***70,777*** | ***77,870*** | ***88,085*** | ***99,952*** | ***116,915*** | ***141,008*** | ***165,101*** | ***179,301*** | ***199,476*** | ***209,450*** |
| 129 | Timor_Leste | *15* | *15* | *16* | *17* | *18* | *18* | *19* | *20* | *20* | ***21*** | *22* |
| 130 | Togo | *443* | *457* | *512* | ***705*** | ***867*** | ***755*** | ***792*** | ***913*** | ***1,075*** | *1,021* | *1,040* |
| 131 | Tonga | *42* | *40* | *43* | *49* | *56* | *64* | *69* | *77* | *81* | ***74*** | *71* |
| 132 | Tunisia | *1,545* | *1,650* | *1,810* | *2,276* | *2,710* | *2,895* | *3,218* | *3,871* | *4,658* | *4,685* | *5,023* |
| 133 | Turkey | *20,412* | *14,268* | *17,348* | *23,515* | *31,532* | *39,941* | ***49,986*** | ***56,024*** | *64,047* | *52,499* | *61,844* |
| 134 | Turkmenistan | *790* | *1,142* | *1,480* | *2,019* | *2,586* | *3,211* | *4,123* | *5,138* | *3,580* | *3,219* | *3,830* |
| 135 | Tuvalu | *6* | *6* | *6* | *6* | *6* | *6* | *6* | *6* | ***6*** | ***6*** | *6* |
| 136 | Uganda | *9,908* | *9,122* | *9,656* | *10,214* | ***18,779*** | ***14,623*** | ***15,804*** | ***19,262*** | ***38,693*** | *26,137* | *29,729* |
| 137 | Ukraine | *6,045* | *7,744* | *8,968* | *11,080* | ***15,134*** | ***21,298*** | ***28,146*** | ***46,798*** | ***59,474*** | *31,405* | *35,016* |
| 138 | Uruguay | ***8,282*** | ***8,553*** | ***7,455*** | ***6,946*** | ***6,438*** | ***6,824*** | ***7,933*** | ***9,906*** | *13,441* | *13,630* | *18,238* |
| 139 | Uzbekistan | *1,151* | *1,095* | *1,024* | *1,243* | *1,723* | *2,384* | *3,231* | *4,781* | *6,760* | ***8,236*** | *9,753* |
| 140 | Vanuatu | *30* | *28* | *28* | *35* | *41* | *45* | *50* | *62* | ***73*** | ***73*** | *85* |
| 141 | Venezuela | ***38,532*** | ***44,545*** | ***36,770*** | ***30,175*** | ***29,243*** | ***39,012*** | ***53,987*** | ***79,730*** | ***99,113*** | ***105,220*** | *92,006* |
| 142 | VietNam | *2,266* | ***4,827*** | ***4,608*** | ***4,390*** | ***5,550*** | ***5,590*** | ***5,738*** | ***7,959*** | ***9,202*** | ***9,590*** | *10,937* |
| 143 | Yemen | *44* | *45* | *49* | *54* | *65* | *80* | *93* | *107* | *137* | ***126*** | *160* |
| 144 | Zambia | *9,578* | *10,894* | *11,297* | *13,141* | *17,020* | ***32,000*** | ***37,403*** | *43,378* | *57,702* | *50,425* | *64,714* |
| 145 | Zimbabwe | *21,909* | *21,894* | *21,819* | ***21,701*** | ***21,557*** | ***19,712*** | ***63,437*** | ***31,896*** | ***15,593*** | ***17,708*** | *21,216* |
